# Impact of Long-Term Endurance Training vs. Guideline-Based Physical Activity on Brain Structure in Healthy Aging

**DOI:** 10.3389/fnagi.2016.00155

**Published:** 2016-06-30

**Authors:** Katelyn N. Wood, Robert Nikolov, J. Kevin Shoemaker

**Affiliations:** ^1^Department of Kinesiology, Neurovascular Research Laboratory, School of Kinesiology, Western UniversityLondon, ON, Canada; ^2^Robarts Research Institute, Western UniversityLondon, ON, Canada; ^3^Department of Physiology and Pharmacology, Western UniversityLondon, ON, Canada

**Keywords:** aerobic exercise, cortical mass, masters athletes, guideline-based fitness

## Abstract

Brain structure is a fundamental determinant of brain function, both of which decline with age in the adult. Whereas short-term exercise improves brain size in older adults, the impact of endurance training on brain structure when initiated early and sustained throughout life, remains unknown. We tested the hypothesis that long-term competitive aerobic training enhances cortical and subcortical mass compared to middle to older-aged healthy adults who adhere to the minimum physical activity guidelines. Observations were made in 16 masters athletes (MA; 53 ± 6 years, VO_2max_ = 55 ± 10 ml/kg/min, training > 15 years), and 16 active, healthy, and cognitively intact subjects (HA; 58 ± 9 years, VO_2max_ = 38 ± 7 ml/kg/min). T1-weighted structural acquisition at 3T enabled quantification of cortical thickness and subcortical gray and white matter volumes. Cardiorespiratory fitness correlated strongly with whole-brain cortical thickness. Subcortical volumetric mass at the lateral ventricles, R hippocampus, R amygdala, and anterior cingulate cortex, correlated with age but not fitness. In a region-of-interest (ROI) group-based analysis, MA expressed greater cortical thickness in the medial prefrontal cortex, pre and postcentral gyri, and insula. There was no effect of group on the rate of age-related cortical or subcortical decline. The current data suggest that lifelong endurance training that produces high levels of cardiorespiratory fitness, builds cortical reserve early in life, and sustains this benefit over the 40–70 year age span. This reserve likely has important implications for neurological health later in life.

## Introduction

Atrophy of cortical gray matter is a hallmark of aging, with tissue loss beginning early in the third decade of life (Good et al., 2001; Raz et al., 2005). Importantly, strong inverse relationships exist between cortical thickness and age-related dementias (Lerch et al., 2005; Du et al., 2007). Furthermore, parallel declines also occur between cognitive function and structural changes in the medial prefrontal cortex, dorso-lateral prefrontal cortex, hippocampus, anterior cingulate, amygdala, and insula (Coffey et al., 1992; Raz et al., 1997, 2005; Good et al., 2001). Given the economic, social, and personal burden associated with age-related neural deterioration, identifying strategic mechanisms to prevent declines in structural and functional brain health *before they begin* represents an imperative public health goal.

Exercise has emerged as a potent stimulus for cognitive improvement in older adults who are sedentary and experience cognitive decline (Voss et al., 1985; Kramer et al., 2005; Chaddock et al., 2012). However, the *extent* to which exercise training can modify cortical thickness or subcortical gray matter volume remains relatively unstudied, with considerable variability across studies. For example, Erickson et al. observed rather rapid increases in hippocampus (HC) volume in just 6 weeks of a walking intervention performed by sedentary, elderly individuals (Erickson et al., 2011). This study shows that exercise is neuroprotective when applied in a late-onset model after atrophy likely has begun, as suggested by cognitive impairment. In a whole brain analysis, Rovio et al. reported that people who were physically active during middle age (~50 years of age) expressed greater frontal lobe cortical thickness at a 21-year follow-up assessment, compared to an inactive control group (Rovio et al., 2010). Similarly, Erikson et al. showed a threshold effect of walking distance over a 9-year follow-up period on cortical thickness in the prefrontal, temporal and hippocampus regions (Erickson et al., 2010). These studies show that activity during the middle age period may exert neuroprotective benefits to a limited portion of the brain later in life. This idea stands in contrast to the knowledge that overall risk of disease and mortality relates to cardiorespiratory fitness (Defina et al., 2013; Barry et al., 2014) or daily energy expenditure (Booth et al., 2012) suggesting that higher gains may be possible than those achieved with mild to moderate interventions such as those provided through published guidelines for active living (ACSM, 1995). Also, the research linking individual differences in physical activity, and training-related changes in fitness to brain health, has led to a hypothesis that cardiorespiratory fitness is a critical mediator of these benefits (Etnier et al., 2006; Angevaren et al., 2008; Smith, 2012). If so, then a dose-response pattern should exist in the building of cortical mass reserve through the life-long pursuit of high fitness levels.

Master's Athletes (MA) provide a unique model to assess the extent to which long-term training can affect brain metrics. Compared to a cohort of inactive elderly controls, Tseng et al. observed greater posterior cortical thickness in the cuneus and precuneus in a small group of older (72 years) MA (Tseng et al., 2013). These results are interesting when considering previous evidence for greater sensitivity of anterior brain regions for age-related brain atrophy (Raz et al., 1997, 2005; Good et al., 2001). The relative lack of exercise effect in these MA in frontal brain regions is of importance because it suggests either a transient effect of exercise on frontal brain mass, or a minimized effect of high training loads on neuroplastic outcomes. Variations in the above studies may also reflect issues such as small sample size, focus on narrow age ranges, varying baseline conditions, short vs. long-term training periods, timing of the exercise onset, and/or emphasis on univariate group-based or region-of-interest (ROI) analysis. In addition, the use of sedentary older individuals as the control condition in many studies introduces a higher probability of undetected nuisance variables such as latent cerebrovascular damage, endothelial dysfunction, and subthreshold neurological impairments. These possibilities raise questions regarding the maximal exercise-induced benefit possible across the adult age span, as well as the appropriate control group for such studies. In addition, it may be that adhering to accepted guidelines for active living (ACSM, 1995) achieves benefits that are not enhanced by additional training loads.

This study attempts to expose the maximal benefit to cortical and subcortical gray matter possible in the context of exercise training. To avoid concerns about subthreshold age-related nuisance variables related to physical inactivity, and to directly relate the differences between guideline-based training vs. high training loads, world-class MA as well as regularly active, but non-competitive, healthy volunteers were studied. We tested the overall hypothesis that gray matter adaptations to exercise follow a dose-dependent pattern in each of the cortical and subcortical gray matter regions.

## Methods

### Participants

A total of 32 individuals participated in this study. Observations were made in 16 elite-level middle-aged and older Masters Athletes (MA) who trained and raced in the sport of triathlon at the professional or national level for >30 years (53 ± 6 years of age (4 female), training >15 h/wk; VO_2max_ = 55 ± 10 ml/kg/min), and 16 similarly-aged healthy, active (HA) control subjects who met the age-appropriate health recommendations for exercise for at least 5 years (58 ± 9 years, (6 female); VO_2max_ = 38 ± 7 ml/kg/min). Table 1 provides group characteristics. All subjects were right-handed, non-smokers, free of medications, and did not have diagnosed hypertension, diabetes, vascular or neurological/psychological impairments. Testing of menstruating females occurred during days 1–14 of the menstrual cycle, with day one representing the first day of menstruation. The postmenopausal women were not taking hormone replacement therapy. The University of Western Ontario Health Sciences Ethics Review Board approved this study and each participant provided informed, written consent. The study adhered to the Declaration of Helsinki.

**Table 1:** **Anthropometric and baseline hemodynamic data (mean ± SD)**.

	**Healthy active**	**Masters athletes**
Age (years)	58 ± 9 Range: 45–73	53 ± 6 Range: 45–67
Resting blood pressure (mmHg)	118/73 ± 9	118/78 ± 9
Resting heart rate (bpm)	58 ± 8	55 ± 9
BMI (kg/m^2^)	26 ± 4	23 ± 3
VO_2max_(mL/kg/min)	38 ± 7 Range: 26–51	55 ± 10^*^ Range: 33–67
Age-predicted VO_2max_(%)	94 ± 19	125 ± 26^*^
LVM (g)	64 ± 14	77 ± 17^*^
Education (years)	12 ± 2	10 ± 3
Trail making score A (secs)	31 ± 8	23 ± 10^*^
Trail making score B (secs)	59 ± 21	53 ± 23
MoCA score	28 ± 2	27 ± 2
Fasting glucose (mmol/L)	5.16 ± 0.35	4.89 ± 0.31^*^
hsC-Reactive Protein (mg/L)	1.54 ± 3.22	1.49 ± 1.17
Cholesterol (mmol/L)	4.46 ± 0.70	4.23 ± 0.76
Triglycerides (mmol/L)	0.92 ± 0.45	0.61 ± 0.22^*^
High density lipoprotein (mmol/L)	1.50 ± 0.40	1.53 ± 0.35
Low density lipoprotein (mmol/L)	2.54 ± 0.60	2.42 ± 0.79
HbA1c	0.06 ± 0.002	0.06 ± 0.002
Gray matter volume (mL)	655 ± 51	691 ± 55^*^
White matter hypointensities (mm^3^)	1924 ± 1221	1501 ± 708
Total intracranial volume (mm^3^)	1.6 × 10^6^ ± 1.5 × 10^5^	1.6 × 10^6^ ± 1.6 × 10^5^

*BMI, body mass index; VO_2max_, maximal oxygen consumption; percent of age-predicted VO_2max_ based on ACSM guidelines, 1995; LVM, left ventricular mass at the heart; education in years including high school; MoCA, Montreal Cognitive Assessment (scored out of 30 possible points); hs, high sensitivity. Cardiac output and LVM indexed to body surface area*.

* *Different from healthy active, p < 0.05*.

### Assessment of cardiorespiratory fitness

A graded treadmill exercise test, conducted under standard clinical observation, provided information regarding each subject's peak oxygen uptake (VO_2max_). During this test, analysis of expired air samples occurred over 3-s intervals until the point of volitional exhaustion. Based on the American College of Sports Medicine guidelines (ACSM, 1995), VO_2max_ was determined by meeting at least three of the following criteria: (1) VO_2_ ceased to increase with increasing workloads (plateau); (2) heart rate reached the age-predicted value (220-age); (3) respiratory exchange ratio > 1.0; and (4) blood lactate > 8.0 mmol/L. These methods have been tested and validated extensively in previous studies of older subjects (Levine, 2008; Fujimoto et al., 2010).

### Laboratory data acquisition

Participants completed two separate experimental sessions: (1) physiological laboratory recording, and (2) magnetic resonance neuroimaging session (MRI; Robarts Research Institute Centre for Functional and Metabolic Imaging). The two sessions were performed at the same time of day and separated by a minimum period of 1 week. Participants practiced the experimental procedures prior to their first test session. Participants reported to the laboratory following a 12 h abstinence from nicotine, alcohol, caffeine and intense physical exertion. Venous blood sampling occurred following 30 min of quiet, supine rest. Analysis of these samples assessed and confirmed baseline levels of blood-borne acute phase inflammatory markers (hsCRP) and glycemic status. Heart rate was measured using a standard three-lead electrocardiogram. Finger photoplethysmography (Finometer; Finapres Medical Systems, Amsterdam, The Netherlands) provided continuous measures of arterial blood pressure following calibration to manual sphygmomanometer blood pressure measurements obtained throughout the protocol. Data were collected using LabChart7 and PowerLab data acquisition system (ADInstruments). Neurological screening was also completed during the laboratory session to exclude dementia (The Montreal Cognitive Assessment) and confirm intact executive function (Trail making tests A and B).

### Neuroimaging data acquisition

Participants completed a structural MRI session following a 12 h abstinence from nicotine, alcohol, caffeine and intense physical exertion. All imaging data were collected using a whole body 3-Tesla imaging system (Magnetom Prisma, Siemens Medical Solutions, Erlangen, Germany) with a 32-channel head coil (Barberi et al., 2000). A high-resolution T1-weighted structural volume was acquired with a 3D MPRAGE sequence at the beginning of the scanning session (sagittal, matrix 256 × 240 mm, voxel resolution 1.0 × 1.0 × 1.0 mm, 1 mm slice thickness, no gap, flip angle 9⋅, TE = 2.98 ms, TI = 900 ms, TR = 2.3 ms). Head movement was limited during the experimental session within a head cradle packed with foam padding, and each subject received instruction to avoid head movements during the scanning period.

### Neuroimaging data analysis

We used the analysis approaches best suited to measure gray and white matter cortically and subcortically, from high-resolution MRI images.

### Subcortical level

Semiautomatic software quantified ventricular and subcortical volumes, as well as total intracranial volume, total gray matter and white matter hypointensities (Freesurfer Image Analysis Suite, http://surfer.nmr.mgh.harvard.edu/). The technical details of these procedures are described in prior publications (Dale and Sereno, 1993; Dale et al., 1999; Fischl and Dale, 2000; Fischl et al., 2001, 2002, 2004). Briefly, this processing included motion correction and averaging (Reuter et al., 2012) of T1-weighted images, removal of non-brain tissue (Ségonne et al., 2004), automated Talairach transformation, segmentation of the subcortical white matter and deep gray matter volumetric structures of interest (including hippocampus, amygdala, anterior cingulate cortex, lateral ventricles) (Fischl et al., 2002, 2004), intensity normalization (Sled et al., 1998), delineation of the gray and white matter boundary, automated topology correction (Fischl et al., 2004; Ségonne et al., 2004) and surface deformation following intensity gradients for optimal placement of gray/white and gray/cerebrospinal fluid borders at the location where the greatest shift in intensity defines the transition to the other tissue (Dale and Sereno, 1993; Dale et al., 1999; Fischl and Dale, 2000). Measurement of ventricular volume is particularly amenable to segmentation due to the high signal intensity contrast between cerebrospinal fluid and surrounding brain tissue in T_1_-weighted MRI. Of note, ventricle volume segmentation included only the lateral ventricles. Adjusting regional volumes to intracranial volume accounted for the potential impact of sex and height on structural outcomes. Subcortical results were corrected for multiple comparisons at FDR, *p* < 0.05.

Cognitive and age-related cortical decline appear to overlap in cortical sites such as the medial prefrontal cortex and insula; as well at specific subcortical sites including the hippocampus, anterior cingulate, and amygdala (Coffey et al., 1992; Raz et al., 1997, 2005; Good et al., 2001). Thus, we used a ROI analysis to focus on these most vulnerable deep gray matter sites (hippocampus, amygdala and anterior cingulate) as well as the fluid-filled lateral ventricles (Good et al., 2001; Resnick et al., 2003; Raz et al., 2005, 2010; Kennedy et al., 2009). White-matter hypointensities provided an index of cerebrovascular disease and cognitive decline (Debette and Markus, 2010). In this case, Freesurfer provides reliable sensitivity in measuring white matter damage in non-demented older adults (Leritz et al., 2014).

### Cortical level

Cortical thickness analysis was performed using Brain Voyager 2.8.4 (BVQX, Brain Innovation, Maastricht, Netherlands). The Lapalace method provided measures of cortical thickness for each subject's right and left hemisphere (Jones et al., 2000). An inter-subject cortical alignment procedure reduced the effect of anatomical variability and improved the spatial correspondence of cortical areas between individual brains. Spatial intensity inhomogeneities in the original T_1_-weighted scans were corrected, converted into volumes with 1-mm isotropic voxel resolution using sinc-interpolation, and transformed spatially into standard Talairach space. Once normalized, an automatic segmentation of white-matter and gray-matter boundaries was applied, and images were resampled to 0.5 mm isovoxel resolution. Manual correction removed topological errors such as “bridges” and remaining fragments of dura mater or cerebellum on a slice-by-slice and individual basis. The reconstructed cortical hemispheres were morphed into a folded three-dimensional mesh. Cortex-based-alignment followed, producing a spherical representation of the folded cortex and finally for each hemisphere. The curvature information of individual brains enabled cortex-based inter-subject alignment (Goebel et al., 2006), and resulted in average curvature maps for each hemisphere, for each group. Each mesh resulted from averaging 16 individual datasets (16 MA and 16 HA).

### Statistical analysis

A two-tailed unpaired *t*-test assessed group-level hemodynamic and anthropometric characteristics, as well as the effect of group on cortical thickness (*p* < 0.05; Systat Software 12.5, 2011). Data are presented as mean ± standard deviation. Overlap existed in the fitness levels of the MA and HA group members. Therefore, following a combining of groups, multiple linear regression analysis assessed the independence of the relationship between subcortical volumes and cortical thickness after adjusting for age, VO_2max_, body mass index (BMI), and left cardiac ventricular mass (indexed to body surface area), as they exert strong effects on the morphology of the cortex (Table 2; Barnes et al., 2003). A threshold for significance was set at *p* < 0.05. All regression analyses passed the normality test, as assessed by the Shapiro-Wilk test for normal distribution. The effect of high cardiorespiratory fitness on the age-related decay of cortical and subcortical mass at the specified regions of interest was determined using linear regression and was compared using Student's *t*-test.

**Table 2:** **Whole-brain subcortical volumes**.

**Subcortical Site**	**Mean MA (mm^3^)**	**Mean HA (mm^3^)**	**Difference (MA-HA)**	***P*-value**	**Corrected *P*-value**
Lateral ventricles	0.0119	0.0136	−0.0017	0.41	0.55
Brain stem	0.0301	0.0140	0.0161	0.27	0.54
CSF	0.0477	0.0007	0.0470	0.71	0.74
L Cerebellum WM	0.0097	0.0092	0.0005	0.15	0.38
R Cerebellum WM	0.0276	0.0094	0.0181	0.29	0.54
L Cerebellum C	0.0367	0.0320	0.0047	0.14	0.38
R Cerebellum C	0.0603	0.0338	0.0265	0.47	0.58
L Thalamus	0.0464	0.0054	0.0411	0.63	0.68
R Thalamus	0.0197	0.0045	0.0152	0.24	0.54
L Caudate	0.0236	0.0021	0.0215	0.33	0.54
R Caudate	0.0085	0.0022	0.0063	0.09	0.38
L Putamen	0.0471	0.0030	0.0441	0.11	0.38
R Putamen	0.0210	0.0029	0.0182	0.28	0.54
L Pallidum	0.0273	0.0008	0.0264	0.39	0.55
R Pallidum	0.0078	0.0010	0.0068	0.10	0.38
L Hippocampus	0.0342	0.0026	0.0316	0.48	0.58
R Hippocampus	0.0097	0.0020	0.0077	0.12	0.38
L Amygdala	0.0034	0.0009	0.0024	0.03^*^	0.35
R Amygdala	0.0109	0.0010	0.0099	0.15	0.38
L Accumbens	0.0014	0.0002	0.0012	0.02^*^	0.35
R Accumbens	0.0235	0.0003	0.0232	0.35	0.54
L Ventral DC	0.0046	0.0023	0.0024	0.04^*^	0.35
R Ventral DC	0.0053	0.0022	0.0031	0.05^*^	0.35
Posterior CC	0.0256	0.0006	0.0250	0.38	0.55
Mid-Posterior CC	0.0497	0.0003	0.0494	0.74	0.74
Central CC	0.0358	0.0003	0.0355	0.53	0.59
Mid-Anterior CC	0.0225	0.0003	0.0223	0.33	0.54
Anterior CC	0.0347	0.0006	0.0342	0.51	0.59

*All values indexed to total intracranial volume. L, left; R, right; WM, white matter; GM, gray matter; C, cortex; DC, dorsal column; CC, cingulate cortex*.

* *Different from HA, p < 0.05*.

To assess the effects of fitness and age on regional brain tissue health, we performed a full cortex correlation analysis in Brain Voyager using each individual's VO_2max_ and age in years. The final correlation maps were adapted by switching off the positive or negative correlation values respectively, increasing the minimum threshold value (*p* < 0.005), and adding a cluster threshold (10 mm^2^).

## Results

Table 1 provides descriptive group information. Participant ages ranged from 45 to 73 years in HA and 45 to 67 years in MA, with a mean age of 58 and 53 years, respectively. Overall, the sample was 31% female. Groups did not differ with respect to age, sex, years of education, blood pressure, heart rate and/or BMI. The HA group demonstrated near 100% of age-predicted VO_2max_, consistent with self-report confirmation of adherence to age-appropriate physical activity guidelines (ACSM, 1995). Compared to HA participants, MA had a greater VO_2max_, and greater left cardiac ventricular mass. The MoCA and Trail Making Test B scores were not different between groups. HA participants expressed a slightly longer time to completion in Trail Making Test A. Plasma glucose, circulating triglycerides, and systolic blood pressures were greater in HA than MA, but all values were within healthy ranges. Circulating hsCRP and lipid levels, as well as total brain volume indicators, were similar between groups.

### Subcortical gray matter

When indexed to total intracranial volume, average subcortical gray matter volumes were not different between HA and MA (Table 2).

#### Region-of-interest analysis

Average volumes at the lateral ventricles, anterior cingulate cortex, the right and left hippocampus, and the right amygdala were similar in the MA and HA groups (Table 2). However, the left amygdala was larger in MA vs. HA (*p* < 0.05).

#### Covariate analysis

Pearson correlation analysis revealed that ventricle volume correlated inversely, albeit weakly, with VO_2max_ (*r* = −0.28, *p* = 0.05). Multiple linear regression revealed age as the strongest predictor of lateral ventricle volume (*p* < 0.001), right hippocampus (*p* < 0.05), right amygdala (*p* < 0.05), and anterior cingulate cortex volumes (*p* < 0.05, Table 3).

**Table 3:** **Results of multiple linear regression of ROI subcortical volumes against anthropometric covariates**.

		**Std. Coefficient**	**Std. Error**	***P*-value**	**Power (α 0.05)**
Lateral ventricles	Age	0.000483	0.000138	**0.001**	0.997
	VO_2max_	0.000146	0.000117	0.218	–
	Body Mass Index	−0.000273	0.000277	0.329	–
	L Ventricular Mass	−0.000122	0.000058	0.041	–
L Hippocampus	Age	−0.000016	0.000009	0.072	–
	VO_2max_	−0.000004	0.000008	0.597	–
	Body Mass Index	0.000006	0.000018	0.751	–
	L Ventricular Mass	−0.000002	0.000004	0.675	–
R Hippocampus	Age	−0.000015	0.000008	**0.049**	0.894
	VO_2max_	0.000002	0.000006	0.705	–
	Body Mass Index	0.000019	0.000015	0.184	−
	L Ventricular Mass	−0.000005	0.000003	0.135	–
L Amygdala	Age	−0.000003	0.000003	0.373	–
	VO_2max_	0.000005	0.000002	0.073	–
	Body Mass Index	0.000003	0.000006	0.606	–
	L Ventricular Mass	−0.000001	0.000001	0.349	–
R Amygdala	Age	−0.000008	0.000003	**0.009**	0.937
	VO_2max_	−0.000001	0.000002	0.742	–
	Body Mass Index	0.000008	0.000006	0.173	–
	L Ventricular Mass	−0.000001	0.000001	0.414	–
Anterior CC	Age	−0.000005	0.000002	**0.033**	0.675
	VO_2max_	−0.000002	0.000002	0.249	–
	Body Mass Index	0.000001	0.000004	0.872	–
	L Ventricular Mass	−0.000001	0.00001	0.433	–

*All individuals were entered into regression, irrespective of group. L ventricular mass indexed to body surface area. Std, Standardized; L, left; R, right; CC, cingulate cortex. Bold typeface, p < 0.05*.

**Figure 1:**
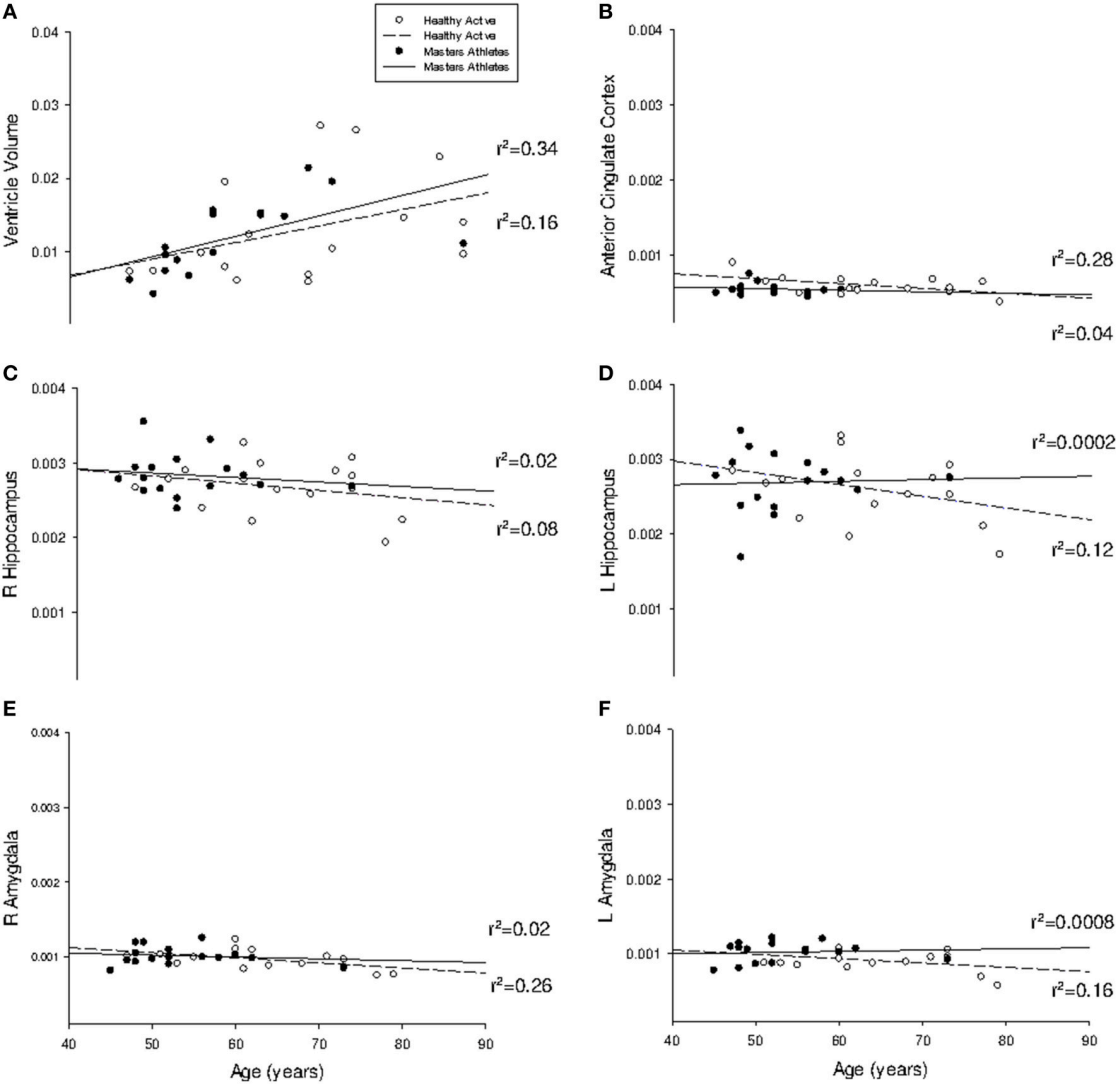
**Regression analysis of region-of-interest subcortical volumes with age**. Slope of regression was not different between groups for **(A)** lateral ventricle volume (probability = 0.75); **(B)** anterior cingulate cortex (probability = 0.31); **(C)** right hippocampus (probability = 0.57); **(D)** left hippocampus (probability = 0.25); **(E)** right amygdala (probability = 0.44); and **(F)** left amygdala (probability = 0.11). All volumes are indexed to total intracranial volume and measured in mm^3^.

The mean rate of ventricular volume enlargement was 0.03% per year in HA and 0.04% per year in MA (*p* = 0.75; Figure 1). The mean annual rate of atrophy at the anterior cingulate cortex was −0.0006% in HA and −0.0002% in MA, which was not different between groups (*p* = 0.31). The mean rate of atrophy at the right hippocampus (HA: −0.0009%; MA: −0.0006%) was not different between groups (*p* = 0.57), nor was it different at the left hippocampus (*p* = 0.25) in either HA (−0.0016%) or MA (0.0002%). The right amygdala also showed no difference in rate of volume change (*p* = 0.44) in either HA (−0.0007%) or MA (−0.0003%), which was consistent with the left amygdala (*p* = 0.11; HA: −0.0006%; MA: 0.0002%). Pearson Correlation analysis confirmed linear dependence between age and ventricle volume (*r* = 0.49, *p* < 0.001), and an inverse correlation between age and VO_2max_ (*r* = −0.71, *p* < 0.001).

### Cortical gray matter

**Figure 2:**
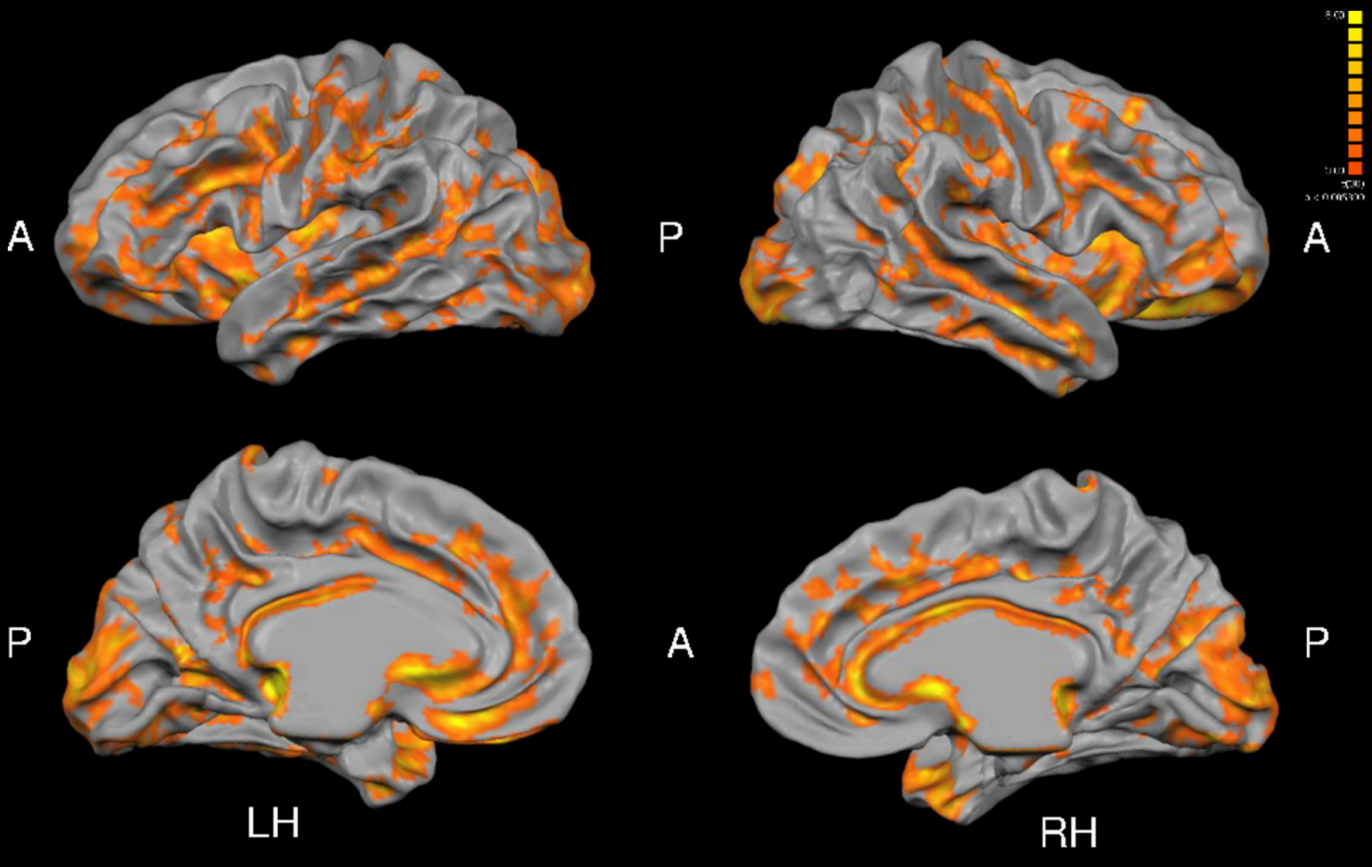
**Group differences in cortical thickness (MA > HA)**. Results are shown at *p* < 0.005, corrected by cluster size (10 mm^2^). Color scale at right denotes *t*-values. LH, left hemisphere; RH, right hemisphere; A, anterior; P, posterior. Many regions in MA were significantly thicker than HA subjects (see Tables 5, 6).

Total gray matter was greater in MA than in HA (*p* < 0.05). An unpaired *t*-test revealed group differences in cortical thickness throughout a wide range of cortical areas (*p* < 0.005, corrected for multiple comparisons by cluster threshold = 10 voxels; Figure 2, Tables 4, 5). The most prominent differences (>1.5 mm) were observed in occipital and temporal regions. Smaller clusters of group differences in cortical thickness (>1 mm) were also detected within the frontal lobe and medial plane. Additionally, there was a dominant lateralization pattern to the left hemisphere, which had greater cortical thickness in many regions, than the right hemisphere.

**Table 4:** **Right hemisphere brain regions showing significant group differences in cortical thickness in a whole-brain analysis**.

**Right hemisphere region**	**Mean MA (mm)**	**Mean HA (mm)**	**Difference (MA-HA)**	**Std. Error**	***t*-value**
Calcarine S	3.1	2.6	0.5	0.13	3.80
Central S	2.3	2.0	0.3	0.04	8.60
Cingulate G	3.2	2.7	0.5	0.08	5.40
Cingulate S	2.7	2.4	0.3	0.04	7.30
Collateral S	3.5	2.8	0.7	0.14	5.30
Cuneus	2.4	2.0	0.4	0.06	6.40
Rectus G	3.0	2.8	0.2	0.09	2.20
Inf FG	2.7	2.4	0.3	0.06	5.70
Inf FS	2.8	2.3	0.5	0.09	5.60
Inf OG	3.1	2.4	0.7	0.12	6.00
Inf PL	2.7	2.3	0.4	0.07	5.60
Inf TG	3.5	2.9	0.6	0.09	7.10
Inf TS	3.3	2.8	0.5	0.10	5.40
Insula	3.4	2.7	0.7	0.07	9.00
Intraparietal S	2.5	2.2	0.3	0.05	5.60
Lat OTG	3.5	2.9	0.6	0.11	5.30
Lat S	2.7	2.4	0.3	0.04	8.50
Med OTG	2.8	2.3	0.5	0.10	5.20
Middle FG	2.6	2.3	0.3	0.05	6.80
Middle OG	2.9	2.3	0.6	0.09	6.50
Middle TG	3.0	2.6	0.4	0.08	5.00
OTS	3.5	2.9	0.6	0.14	4.20
Olfactory S	3.9	2.5	1.4	0.24	5.90
Orbital G	3.5	2.7	0.8	0.11	7.10
Orbital S	3.2	2.5	0.7	0.10	6.70
Parahippocampal G	4.1	3.3	0.8	0.18	4.30
POS	2.5	2.2	0.3	0.05	7.30
Postcentral G	2.2	1.9	0.3	0.04	7.80
Postcentral S	2.4	2.1	0.3	0.05	6.70
Precentral G	2.4	2.1	0.3	0.04	5.80
Precentral S	2.6	2.2	0.4	0.05	7.20
Precuneus	2.7	2.3	0.4	0.06	5.30
Sup FG	2.7	2.5	0.2	0.04	5.20
Sup FS	2.7	2.3	0.4	0.04	7.70
Sup OG	2.8	2.2	0.6	0.10	6.10
Sup PL	2.3	2.1	0.2	0.04	5.50
Sup TG	2.7	2.5	0.2	0.05	4.40
Sup TS	3.0	2.5	0.5	0.07	6.20
Supramarginal G	2.7	2.4	0.3	0.05	6.00
Trans OS	2.6	2.2	0.4	0.07	4.70
Med PreF	3.9	3.1	0.8	0.17	4.50

*MA, masters athletes; HA, healthy active. S, sulcus; G, gyrus; Inf, inferior; Lat, lateral; Med, medial; Sup, superior; Trans, transverse; F, frontal; O, occipital; P, parietal; T, temporal; L, lobe. All p < 0.005*.

**Table 5:** **Left hemisphere brain regions showing significant group differences in cortical thickness in a whole-brain analysis**.

**Left hemisphere region**	**Mean MA (mm)**	**Mean HA (mm)**	**Difference (MA-HA)**	**Std. Error**	***t*-value**
Calcarine S	2.9	2.0	0.9	0.14	6.60
Central S	3.2	1.8	1.4	0.16	8.80
Cingulate G	3.9	2.7	1.2	0.14	8.60
Cingulate S	3.8	2.4	1.4	0.22	6.30
Collateral S	4.7	2.9	1.8	0.25	7.10
Cuneus	4.3	2.1	2.2	0.37	6.00
Rectus G	4.4	2.6	1.8	0.23	7.30
Inf FG	2.7	2.2	0.5	0.08	6.30
Inf FS	2.8	2.3	0.5	0.10	5.80
Inf OG	3.0	2.2	0.8	0.14	5.60
Inf PL	2.7	2.2	0.5	0.09	5.90
Inf TG	4.2	2.9	1.3	0.23	6.10
Inf TS	3.3	2.5	0.8	0.12	6.70
Insula	3.2	2.2	1.0	0.10	9.70
Intraparietal S	2.8	2.1	0.7	0.13	5.60
Lat OTG	3.9	2.8	1.1	0.39	2.90
Lat S	2.7	2.2	0.5	0.08	7.20
Med OTG	2.6	1.8	0.8	0.12	6.00
Middle FG	2.8	2.3	0.5	0.08	7.30
Middle OG	3.0	1.9	1.1	0.18	6.40
Middle TG	3.5	2.3	1.2	0.17	7.20
OTS	4.2	2.6	1.6	0.39	4.00
Olfactory S	3.6	2.6	1.0	0.62	1.70
Orbital G	3.9	2.1	1.8	0.26	6.80
Orbital S	3.7	2.7	1.0	0.18	5.40
Parahippocampal G	4.0	3.2	0.8	0.58	1.40
POS	2.5	2.0	0.5	0.08	5.70
Postcentral G	2.7	2.1	0.6	0.06	9.70
Postcentral S	2.5	1.9	0.6	0.09	6.50
Precentral G	3.0	2.1	0.9	0.13	6.20
Precentral S	2.8	2.2	0.6	0.08	6.60
Precuneus	3.5	2.5	1.0	0.17	5.70
Sup FG	3.3	2.4	0.9	0.08	10.00
Sup FS	3.0	2.3	0.7	0.12	5.90
Sup OG	3.0	1.9	1.1	0.18	6.40
Sup PL	2.4	2.0	0.4	0.07	5.90
Sup TG	2.8	2.2	0.6	0.10	6.00
Sup TS	3.8	2.3	1.5	0.22	6.70
Supramarginal G	2.6	2.1	0.5	0.09	5.70
Trans OS	3.3	2.2	1.1	0.18	5.90
Med PreF	3.9	2.8	1.1	0.20	5.60

*MA, masters athletes; HA, healthy active. S, sulcus; G, gyrus; Inf, inferior; Lat, lateral; Med, medial; Sup, superior; Trans, transverse; F, frontal; O, occipital; P, parietal; T, temporal; L, lobe. All p < 0.005*.

#### Region-of-interest analysis

**Figure 3:**
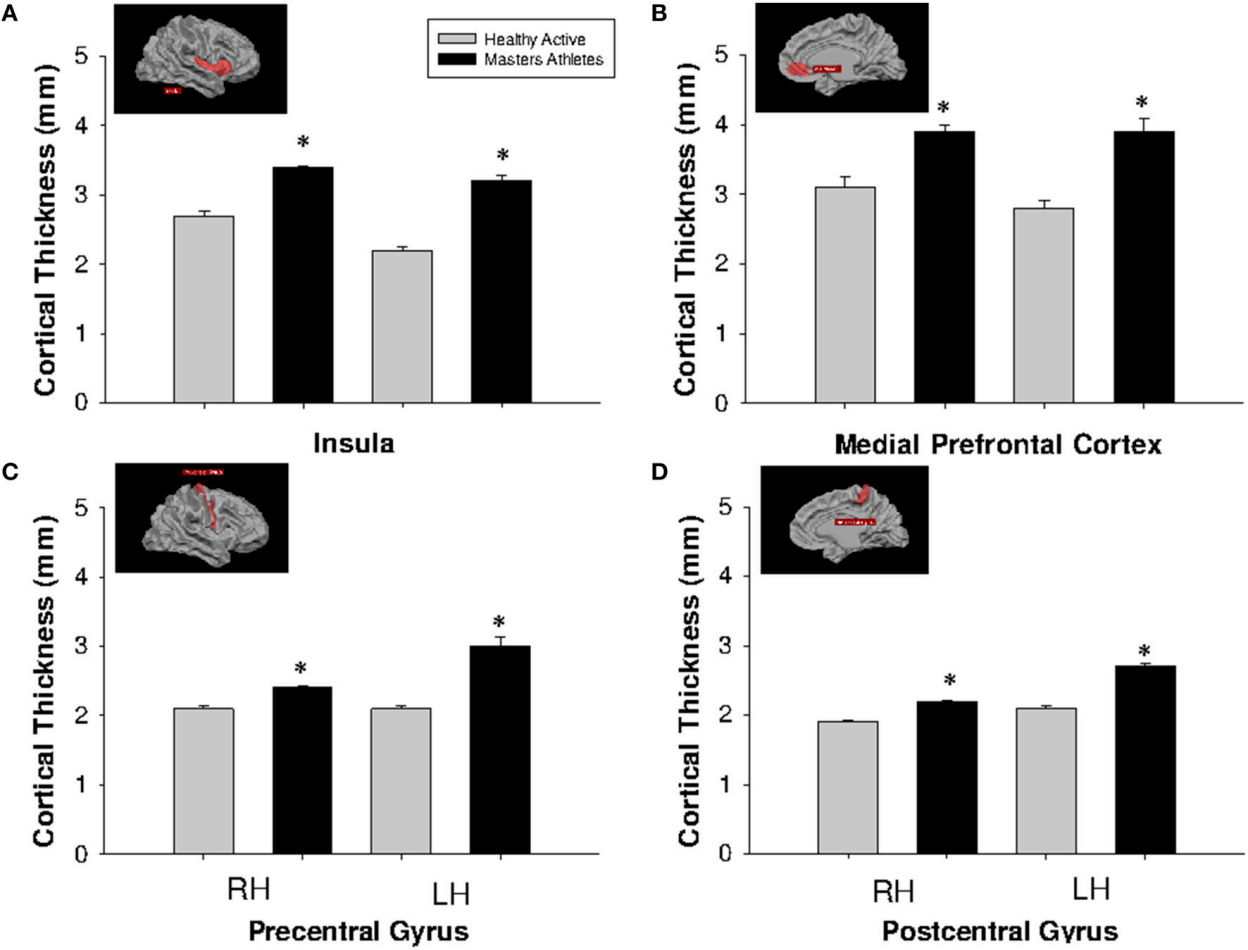
**Cortical thickness at anatomically defined regions of interest in insula (A), medial prefrontal cortex (B), precentral gyrus (C), and postcentral gyrus (D)**. HA (gray bars), MA (black bars). RH, right hemisphere (left side pair of bars); LH, left hemisphere (right side pair of bars). ^*^Different from HA, *p* < 0.0005, corrected by cluster size (10 mm^2^).

Cortical thickness was greater in MA than HA at each region of interest including the bilateral insula, medial prefrontal cortex, precentral and postcentral gyri (Figure 3).

### Covariate analysis

**Figure 4:**
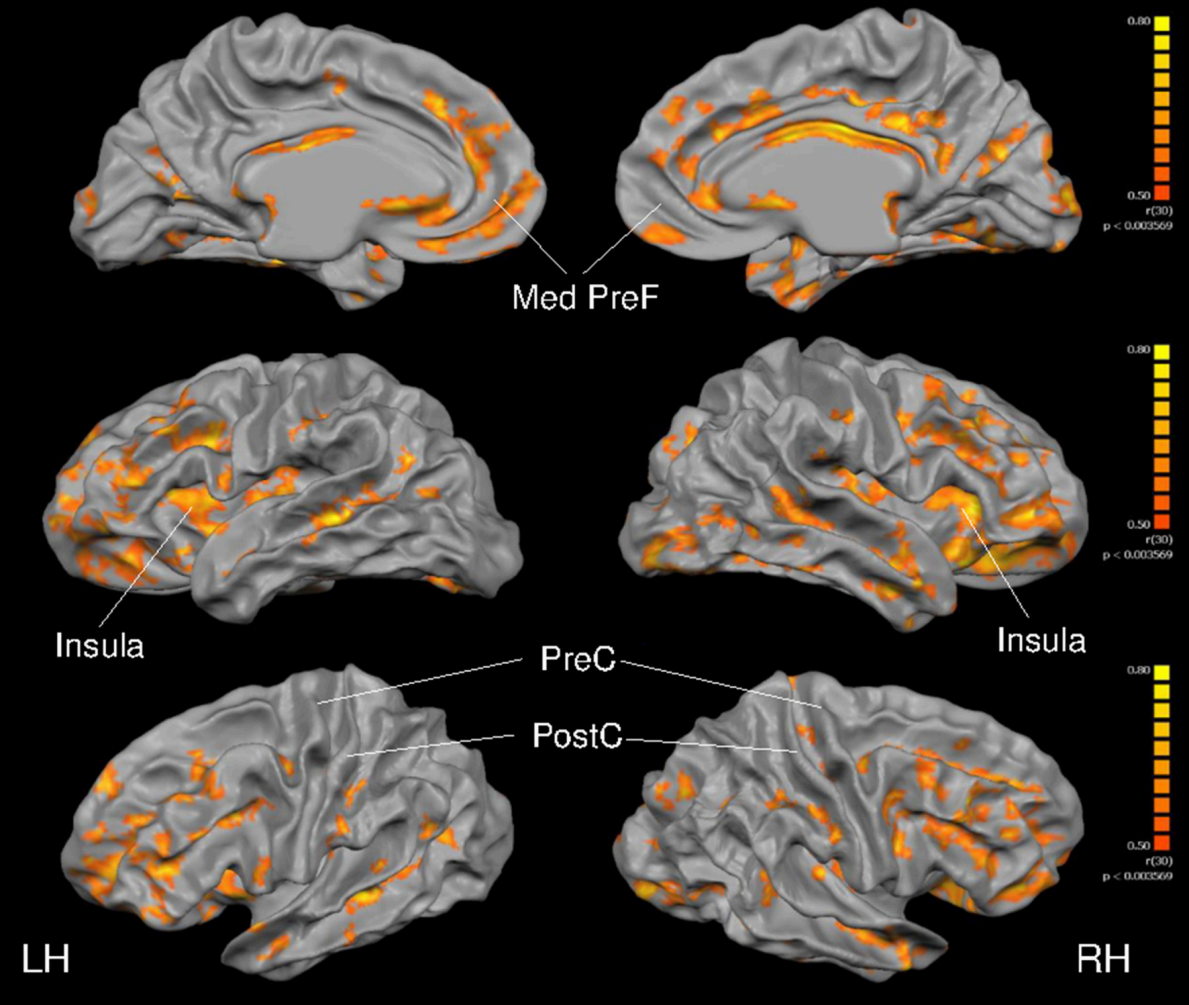
**Full cortex correlation analysis with VO_2max_ covariate**. All subjects, irrespective of group. Results are shown at *p* < 0.005, corrected by cluster size (10 mm^2^). Color scale at right denotes *t*-values. LH, left hemisphere; RH, right hemisphere; Med, medial; PreF, prefrontal; C, central. Regions of interest are labeled.

As depicted in Figure 4, cortical thickness and VO_2max_ exhibited strong correlations across much of the cerebral cortex. Specific results from the multiple linear regression indicated that across all regions of interest, VO_2max_ was the strongest predictor of cortical thickness (Table 6) with the exception of the right precentral gyrus and left postcentral gyrus.

**Table 6:** **Results of multiple linear regression of ROI cortical thickness against anthropometric covariates**.

		**Std. Coefficient**	**Std. Error**	***P*-value**	**Power (α 0.05)**
R Insula	Age	−0.0014	0.008	0.862	–
	VO_2max_	0.0169	0.007	**0.031**	0.999
	Body Mass Index	−0.0116	0.015	0.453	–
	L Ventricular Mass	0.0016	0.004	0.682	–
L Insula	Age	0.0009	0.011	0.935	–
	VO_2max_	0.0318	0.010	**0.005**	0.999
	Body Mass Index	0.0024	0.021	0.911	–
	L Ventricular Mass	−0.0035	0.005	0.515	–
R Med PreF	Age	−0.0008	0.019	0.968	–
	VO_2max_	0.0353	0.018	**0.047**	0.973
	Body Mass Index	0.0198	0.036	0.591	–
	L Ventricular Mass	0.0001	0.009	0.991	–
L Med PreF	Age	0.0134	0.009	0.133	–
	VO_2max_	0.0227	0.008	**0.008**	0.999
	Body Mass Index	−0.0031	0.016	0.852	–
	L Ventricular Mass	0.0070	0.004	0.096	−
R Precentral	Age	0.0007	0.006	0.903	–
	VO_2max_	0.0044	0.005	0.420	–
	Body Mass Index	−0.0094	0.011	0.406	–
	L Ventricular Mass	0.0006	0.003	0.829	–
L Precentral	Age	−0.0002	0.004	0.964	–
	VO_2max_	0.0087	0.004	**0.035**	0.993
	Body Mass Index	0.0008	0.008	0.920	–
	L Ventricular Mass	−0.0033	0.002	0.117	–
R Postcentral	Age	0.0019	0.004	0.647	–
	VO_2max_	0.0083	0.004	**0.039**	0.998
	Body Mass Index	−0.0097	0.008	0.230	–
	L Ventricular Mass	−0.0013	0.002	0.528	–
L Postcentral	Age	0.0041	0.005	0.374	–
	VO_2max_	0.0064	0.004	0.140	–
	Body Mass Index	−0.0069	0.009	0.434	–
	L Ventricular Mass	0.0009	0.002	0.687	–

*All individuals were entered into regression, irrespective of group. L ventricular mass indexed to body surface area. Std, Standardized; L, left; R, right. Bold typeface, p < 0.05*.

**Figure 5:**
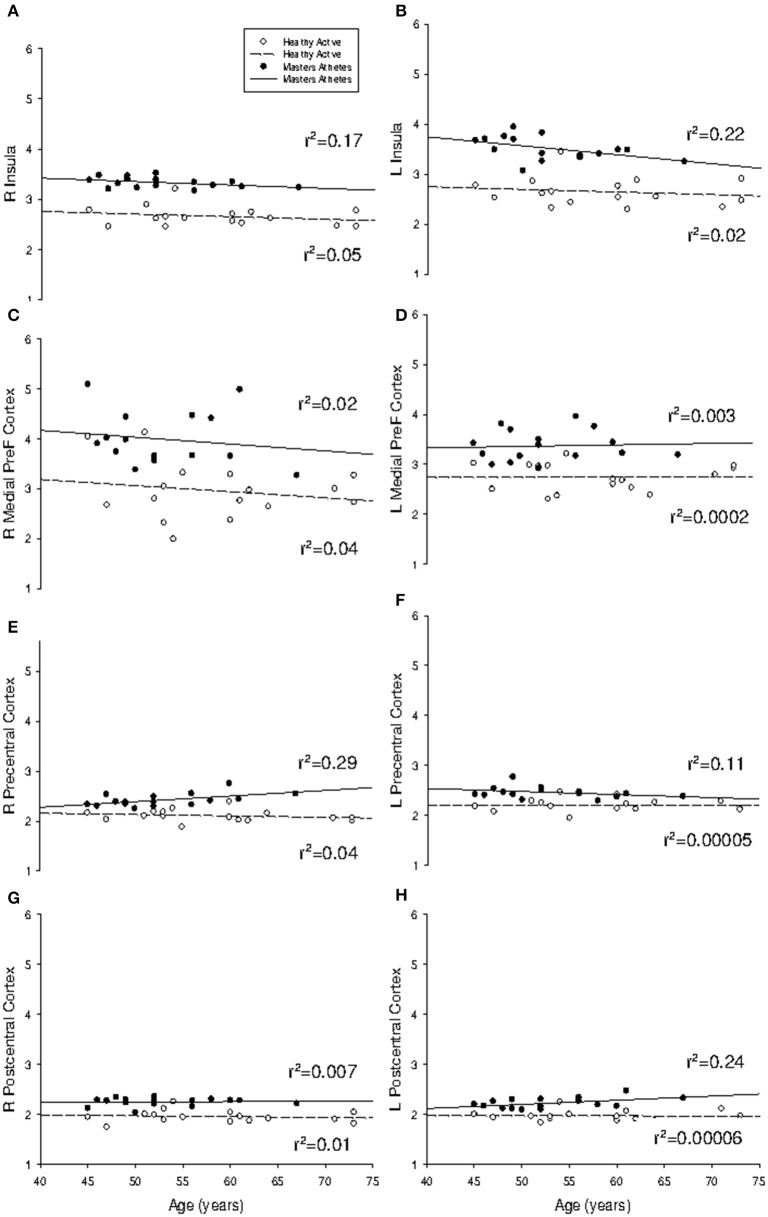
**Regression analysis of region-of-interest cortical thickness with age**. Slope of regression was not different between groups for **(A)** right insula (*P* = 0.82); **(B)** left insula (*P* = 0.32); **(C)** right medial prefrontal cortex (*P* = 0.96); **(D)** left medial prefrontal cortex (*P* = 0.88); **(F)** left precentral gyrus (*P* = 0.33); **(G)** right postcentral gyrus (*P* = 0.95); and **(H)** left postcentral gyrus (*P* = 0.11). Masters athletes had a different trajectory with age at **(E)** right precentral gyrus (*P* = 0.02). Cortical thickness measured in mm.

Of interest, the annual rate of cortical atrophy across the age span (Figure 5) was not different between HA and MA at the right insula (HA: −0.53%; MA: −0.69%; *p* = 0.82), left insula (HA: −0.52%; MA: −1.79%; *p* = 0.32), the right medial prefrontal cortex (HA: −1.23%; MA: −1.37%; *p* = 0.96), or the left medial prefrontal cortex (HA: 0.044%; MA: 0.29%; *p* = 0.88). Also, the mean annual rate of change did not differ between groups at the left precentral gyrus (HA: −0.01%; MA: −0.61%; *p* = 0.33), the right postcentral gyrus (HA: −0.17%; MA: 0.11%; *p* = 0.95) and the left postcentral gyrus (HA: −0.009%; MA: 0.82%; *p* = 0.11). The effect of age on cortical thickness at the right precentral gyrus was different between groups (*p* < 0.05) where little change was observed in HA (−0.28%) but a progressive increase in cortical thickness was observed in MA (0.12%).

**Figure 6:**
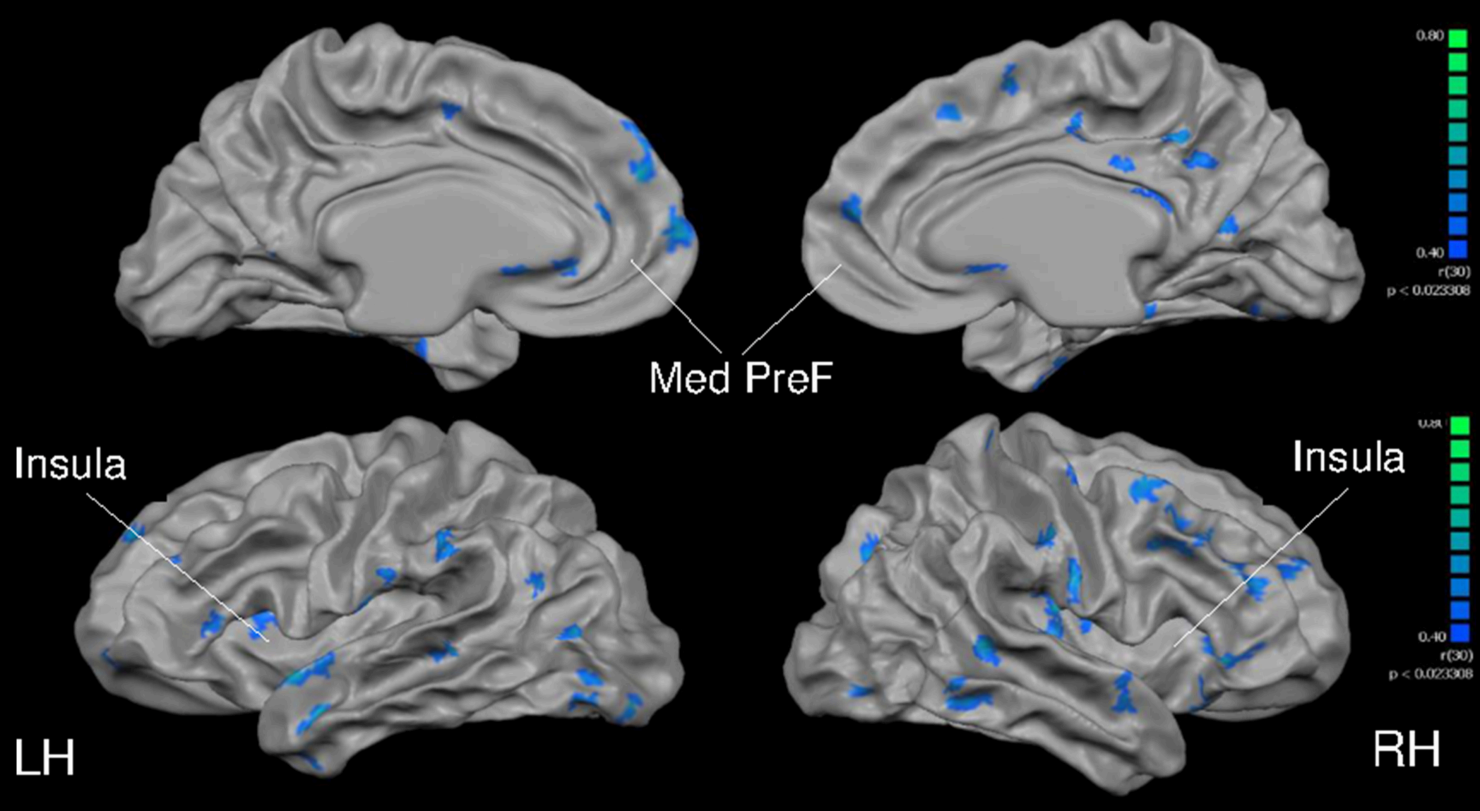
**Full cortex correlation analysis with age covariate**. All subjects, irrespective of group. Results are shown at *p* < 0.05, corrected by cluster size (10 mm^2^). Color scale at right denotes *t*-values. LH, left hemisphere; RH, right hemisphere; Med, medial; PreF, prefrontal. Regions of interest are labeled.

**Figure 7:**
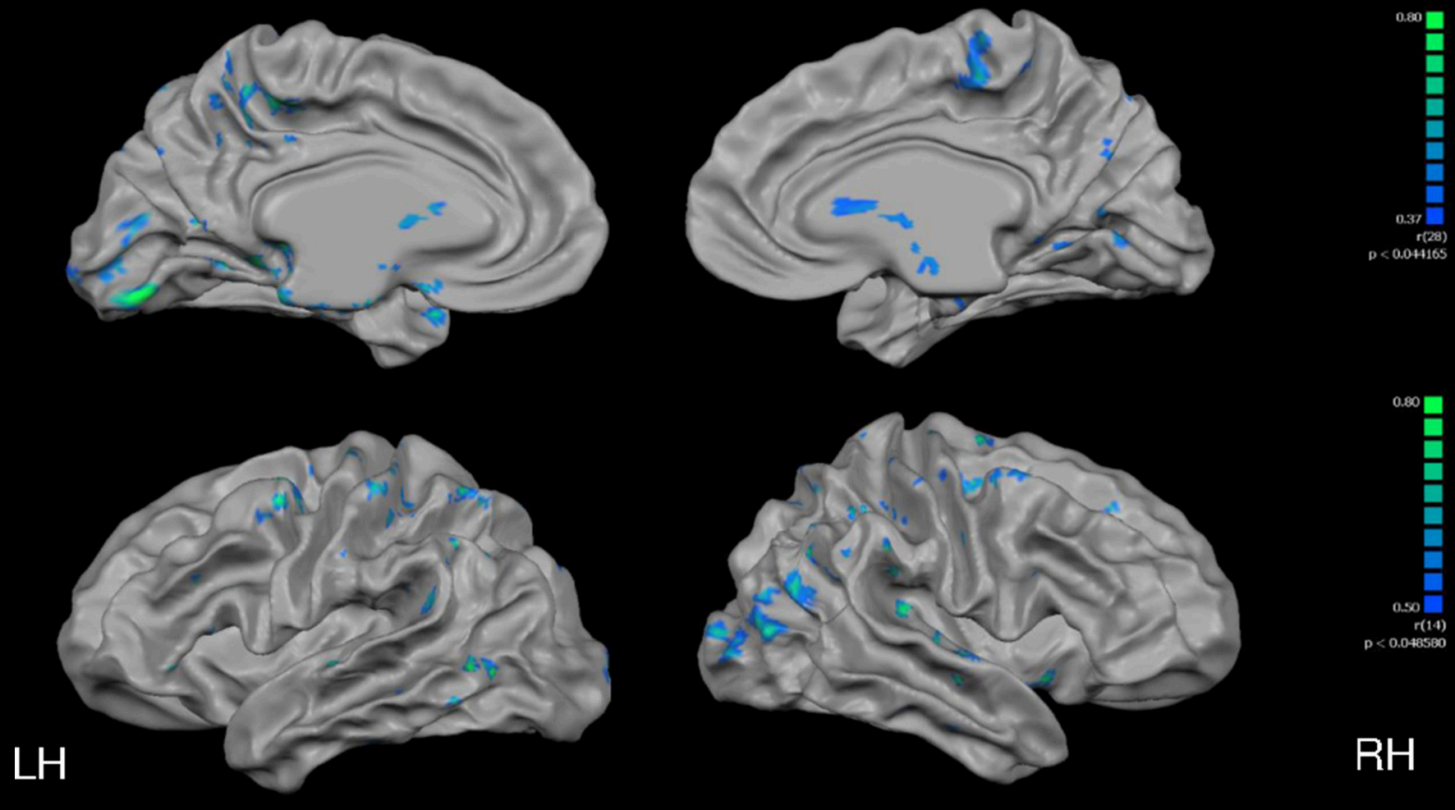
**Full cortex negative correlation analysis with VO_2max_ covariate**. All subjects, irrespective of group. Results are shown at *p* < 0.005, corrected by cluster size (10 mm^2^). Color scale at right denotes *t*-values. LH, left hemisphere; RH, right hemisphere.

In a whole-brain, voxel-wise negative correlation analysis, age correlated negatively with cortical thickness in the parietal, temporal and frontal lobes (Figure 6). Further, a negative correlation analysis revealed regions of cortical thickness that correlated negatively (i.e., was thinner) with VO_2max_ (Figure 7) in MA vs. HA; these regions were few in number and were concentrated around the occipital and parietal cortices.

## Discussion

This study examined the benefit that high levels of exercise training and cardiorespiratory fitness could have upon brain structure in a healthy human model. Overall, long-term training and high levels of cardiorespiratory fitness, elicited and sustained, greater cortical thickness over a large portion of the brain in MA than HA groups. However, life-long exercise training had little effect on the rate of age-related cortical atrophy and, had no effect on subcortical structures relative to that of modestly active adults. In addition, with the possible exception of focal regions of cortical thinning in the occipital and parietal lobes, life-long, high-intensity exercise training did not produce any apparent detrimental effect as indicated by greater total gray matter in MA, equivalent white matter hypointensities, and normal blood-borne inflammatory-markers, when compared to age-matched healthy active counterparts. Therefore, the current results illustrate the potent effect of this very high exercise stimulus to develop cortical (but not subcortical) reserve at younger ages, and sustain this reserve into the senior years. These data suggest that chronic endurance training provides a benefit in cortical neural reserve beyond that provided by guideline-based activity, but does not eliminate the effect of age on cortical gray matter atrophy.

The current study is the first to present evidence that life-long aerobic training, initiated early in life and sustained over decades, has a significant impact on total gray matter, and whole-brain cortical mass. Middle to older-aged MA were chosen for this study as they represent a dedicated athletic career lasting, in some cases, longer than 30 years, a proven athletic performance background, and a high to very high age-adjusted VO_2max_ (~125% of age-predicted maximum). Therefore, a reasonable conjecture is that these individuals represent the highest expected level of exercise training in aging adults and, therefore, provide an index of the potential to grow and preserve brain mass across the age span. Similarly, all HA participants were chosen based on their self-reported history of adherence to a healthy active lifestyle (>5 years) that met the age-adjusted physical activity guidelines (ACSM, 1995). These guidelines emphasize 30 min of moderate intensity exercise on 5 or more days of the week, confirmed by an age-adjusted VO_2max_ of nearly 100%. In addition, our HA sample had normal weight and BMI scores, and hemodynamic values statistically the same to those of MA and all within normal limits. Therefore, all participants in the current study were free of concerns related to sedentary living such as medications, brain pathology and cognitive dysfunction, and represent a model of active, successful aging (Thielke and Diehr, 2012). Therefore, we propose that the current data represent a specific effect of exercise training.

### Subcortical gray matter and lateral ventricular volume

An interplay between subcortical mass and cerebral spinal fluid volume often results in the passive expansion of the lateral ventricles as neighboring gray matter mass declines (Weuve et al., 2004; Podewils et al., 2005). Numerous cross-sectional imaging studies highlight the reduction in hippocampus (HC) volume with age, accompanied by a significant increase in ventricular volume (Gur et al., 1991; Bhatia et al., 1993; Blatter et al., 1995; Förstl et al., 1995; Jack et al., 1997, 2013; Mu et al., 1999; Cash et al., 2015). However, contradictory data also exist regarding age (Mueller et al., 1998; Resnick et al., 2000) and HC volume (Jack et al., 1989; Ohnishi et al., 2001). The current observations support the idea that age predicts lateral ventricular and HC volumes. However, Pearson correlation analysis revealed that ventricular volume also correlated weakly and inversely with VO_2max_ (*r* = −0.28, *p* = 0.05), due possibly to the expansion of the cerebral cortex space in the MA group, discussed below.

The HC is possibly the most commonly researched brain structure in the context of adaptations to exercise training. Previous evidence indicates a high sensitivity of HC volume to the exercise stimulus. For example, Erickson et al. illustrate rapid expansion of HC volume within 6 weeks of the onset of exercise training in older sedentary adults with a baseline state of cognitive impairment (Erickson et al., 2011). This conclusion contrasts with the current observations that indicate little association between cardiorespiratory fitness and HC volume. However, the current participants were all healthy, in the middle age range, and were at, or above, their age-predicted level of cardiorespiratory fitness for active individuals. Therefore, the potential capacity for HC volumetric changes in response to exercise are likely a function of the starting baseline condition. In the current study, we suspect the lack of HC volumetric change between groups was due to a maximal effect achieved by guideline-based exercise training.

In contrast to the hippocampus, left amygdala volumes were greater in MA vs. HA groups and were related to VO_2max_. The specific benefit of this adaptive response to lifelong training could be multifactorial, given the role of this region in the processing of emotion, learning, memory, and autonomic cardiovascular adjustments.

### Cortical gray matter

In contrast to the subcortical gray matter, we observed a widespread enhancement of cerebral cortex gray matter thickness in the MA group compared to the HA group. The impact of training in MA varied across the brain with some regions expressing no benefit while others indicated ~25% greater cortical thickness. These latter regions were evident in the frontal lobe, temporal lobe, insula cortex, parietal lobe and occipital regions. Thus, marked cortical “reserve” was evident in these regions across the entire age span including the middle age stages where the duration of training was less than those in the higher ages.

As mentioned, a notable outcome of the current study was the widespread cortical thickening in MA vs. HA. Our whole brain analysis (Tables 4, 5) reveals a number of cortical sites including the temporal and occipital lobes. Previously, Tseng et al. (2013) reported the confinement of cortical benefits of training in master's athletes to the posterior portion of the brain in the cuneus and precuneus. The current study differs from the previous Tseng et al. (2013) study in the observation of widespread cortical thickening in MA. A wider age range and larger sample size may factor in these between-study differences. Nonetheless, the current results support those of Tseng et al. (2013) in the observation of greater cortical thickness in the cuneus and precuneus when compared to a sedentary elderly group. The possible implications of these observations are not clear but may relate to the functional role of the default mode network in neurocognitive and autonomic processing (Utevsky et al., 2014).

Further, the current data along with those of Tseng et al. (2013), give rise to a possible outcome that the posterior brain not only exhibits resistance to age-related atrophy but also demonstrates sensitivity to exercise stimuli across the age span studied here. Overall, the current observations support a speculation that the additional cortical reserve achieved early in life through high cardiorespiratory fitness will minimize the risk for neurological impairment in senescence and reduce the frailty period of life.

The mechanisms that operate to enhance cortical structure with training remain unknown and are likely multifactorial. In general, axonal, dendritic, and glial processes account for ~50% of gray matter volume whereas vascular (5%), other cell types (20%) and interstitial space (cerebrospinal fluid) account for the remainder (Thomas et al., 2012). Recent evidence highlights the benefits of exercise training on pial vessel number, synaptogenesis (Cotman and Berchtold, 2002), neurogenesis (van Praag et al., 1999), and astrocyte expansion (Saur et al., 2014). To account for the regional ~25% greater cortical thickness in the MA group, some combination of expansion in any or all of these tissue types may be required.

### Negative association of high fitness with brain structure

Chronic exposure to stress, whether it occurs during childhood, adolescence, adulthood or aging, has a negative impact on brain structure (Sapolsky, 1999; Landfield et al., 2007; Lupien et al., 2009). Freund et al. (2012) illustrated the significant, but reversible, reduction in brain volume following the TransEurope footrace that occurs over several weeks. The MA in our study were training >15 h/wk for decades which may amount to a significant level of physical stress. Therefore, concerns arise regarding the potential damage that intensive long-term competitive events may present to the brain, possibly due to persistent inflammatory mechanisms. However, no differences in white matter hypointensities, chronic inflammatory status (i.e., hsCRP levels), or altered lipidemic or glucogenic stress existed between groups. Therefore, little detrimental effect of chronic competitive training on brain structure occurred in the MA. In contrast, Tseng et al. (2013) observed an 83% reduction in white matter hyposintensities in MA compared to sedentary adults. We attribute this discrepancy to the fact that our HA group was not sedentary and thus preserved white matter integrity equally as well as our MA population. If so, then guideline-based training may be sufficient for maximal preservation of white matter integrity and subcortical structure volumes to the same degree as high-intensity long duration training. We did observe isolated regions of cortical thinning in MA relative to HA, located within the parietal and occipital regions. However, whether this observation reflects variations in regional adaptability or cortical damage remains unknown. It is noteworthy, however, that none of the MA group reported neurological cardiovascular problems such as fibrillation, and the regions of thinner cortex in these athletes have not been associated with autonomic cardiovascular function.

## Conclusion

We present novel findings that sustained high levels of cardiorespiratory fitness increase cortical thickness in many brain regions. However, the impact on subcortical gray matter appears to peak at levels of activity that meet the published guidelines for adults of middle to older age. Nonetheless, despite producing greater cerebral cortex thickness, the MA training load had no effect on the trajectory of age-related cortical atrophy. In fact, although based on small sample sizes, the rates of decline in the selected cortical regions of interest in the current study fall within the expected 0–0.5% range for this age group (Salat et al., 2004; Raz et al., 2005), in each of the MA and HA groups. Therefore, the benefits of life-long exercise training were limited to the expression and sustainment of cortical reserve, but not age-related decline. Therefore, in cognitively normal, healthy individuals, the neurological benefit of chronic training appears to include a sustained cortical reserve, producing greater cortical mass and possibly improved neurological function at every age. Furthermore, life-long, high-intensity exercise did not produce a notable detrimental impact on brain structure. The expectations of these observations are that the 25% reserve in cortical thickness that accompanied lifelong training will preserve neurologic function into the senescent years.

## Limitations

Significant limitations of this study include lack of data on the proportional contributions of the exercise effect vs. the environmental, genetic, or social situations on brain structure, that likely change over time.

We acknowledge the small sample size of the current groups and promote caution in interpreting the results. Nonetheless, using a random selection of 75% of our sample indicated that VO_2max_ remained the strongest predictor of cortical thickness and ROI subcortical volume. We have also used a rigorous threshold protocol for the voxel-wise analysis (*p* < 0.005, cluster size > 10 mm^2^) to reduce the probability of false findings which could be associated with a small sample size.

Our study may also be limited by the age-range of our participants being 45–73 years in HA and 45–67 years in MA. This age range does not reflect the older age range in which cortical atrophy becomes more apparent.

Finally, we acknowledge the limitations of the cognitive testing protocol used in this study. We did not attempt to complete a comprehensive analysis of cognitive function. Rather, we collected MoCA and Trail Making questionnaires as a rapid but valid estimate of overall cognitive health.

## Author contributions

KW: Neuroimaging data collection and analysis; primary manuscript author. RN: Subcortical data analysis. KS: Principal Investigator.

## Funding

This work was funded by the Canadian Institutes for Health Research through a Team Grant in Physical Activity, Mobility and Neural Health (Grant #217532), with KS as the nominated Principal Investigator. KS is a Canada Research Chair in the Integrative Physiology of Exercise and Health.

### Conflict of interest statement

The authors declare that the research was conducted in the absence of any commercial or financial relationships that could be construed as a potential conflict of interest.
